# Evaluation of the efficiency and safety of adjuvant chemotherapy alone for patients with advanced endometrial carcinoma: A protocol for systematic review and meta-analysis

**DOI:** 10.1097/MD.0000000000029156

**Published:** 2022-07-15

**Authors:** Jiayi Guo, Siqi Li, Yuping Wu

**Affiliations:** a Department of Obstetrics and Gynecology, Beijing Jishuitan Hospital, Beijing, China.

**Keywords:** endometrial carcinoma, chemotherapy, safety, efficiency

## Abstract

**Background:**

Endometrial cancer is a tumor that affects many women. Essentially, patients who have high-risk endometrial cancer tend to have a disproportionately high rate of metastasis as well as relapse than the general population. Regardless of complete resection, individuals who are having stages III or IV cancer appear to be at substantial risk of recurrence, either locally or distantly. Chemotherapy and radiation therapy are examples of systemic adjuvant treatment. However, the ideal scheduling strategy remains a mystery. Undertaking this research can help in examining the efficacy as well as safety of adjuvant chemotherapy alone in patients with advanced endometrial cancer in the future.

**Methods:**

To recognize all randomized controlled trials evaluating the efficacy and safety of adjuvant chemotherapy alone in those patients with advanced endometrial carcinoma, a comprehensive systematic review along with meta-analysis were undertaken in PubMed, EMBASE, Cochrane Library, Web of Science, Wanfang, China National Knowledge Infrastructure (CNKI), and Chinese Biological Medical Database (CBM). In accordance with traditional Cochrane methodology, 2 independent authors will review search results, choose studies for inclusion, extract study characteristics and outcome data, and examine the risk of bias in the researches they pick. The *P* values and the *I²* statistic shall be employed in determining the levels of heterogeneity. Meanwhile, the heterogeneity will be explored via the use of sensitivity analyses, and the meta-analysis will be conducted utilizing the RevMan 5.3 software package.

**Results:**

Patients with advanced endometrial cancer will benefit from this research since it will offer a high-quality synthesis of existing information on the utilization of adjuvant chemotherapy alone.

**Conclusion:**

The outcomes of the proposed investigation will summarize the current evidence of adjuvant chemotherapy alone for patients with advanced endometrial carcinoma.

## 1. Introduction

Endometrial cancer is a frequent gynecological cancer in advanced nations, affecting an estimated 417,000 people yearly worldwide along with 66,570 new cases per year in the United States alone. Its incidence is predicted to continue to rise in the coming years.^[1,2]^ Because to the differences in lifestyles and geographical locations in industrialized nations, the incidence rate of female genital malignancies is the greatest, and the average age of patients is becoming younger. The amount of abdominal as well as pelvic disease, nodal involvement, histologic subtype, presence of extranodal disease, along with completeness of surgical resection are all clinical and pathologic criteria that influence the likelihood of recurrence.^[3–5]^ Considering that signs of nonconforming genital bleeding manifest themselves in over 90% of patients, most patients are detected in their early stages. The International Federation of Gynecology and Obstetrics estimates that roughly 75% to 80% of endometrial cancer patients are identified when the disease is discovered early. Patients at stage I and stage IA stages tend to present with a fair prognosis, usually with about 5-year survival rate of >90% in these stages.^[6,7]^

Regardless of complete resection, individuals with stage III or IV cancer are at substantial risk of recurrence, either locally or distantly.^[8]^ Chemotherapy and radiation are examples of systemic adjuvant treatment. However, it is not clear what the ideal scheduling strategy is. Over several decades, patients at high-risk of recurrence as well as mortality have been provided adjuvant radiation following surgical intervention.^[9,10]^ Several recent studies have shown that adjuvant radiation had little effect on the survival of individuals with high-risk endometrial cancer.^[9,10]^ Because most endometrial cancer patients die due to extraperitoneal illness, adjuvant chemotherapy has been included to treat patients with locally advanced endometrial cancer or instances with aggressive histological types.^[9,10]^ Many randomized studies have addressed this problem with mixed success.^[9,10]^ The possible advantage of adjuvant chemotherapy in treating advanced endometrial cancer has been investigated in several trials, but the findings have remained disputed.^[11–13]^ For this reason, we will first assess the efficacy and safety of adjuvant chemotherapy alone in patients with advanced endometrial cancer in the current research.

## 2. Objectives

The current research is intended to be carried out to offer a comprehensive assessment of the efficacy and safety of adjuvant chemotherapy alone in patients with advanced endometrial cancer, based on the collection and analysis of relevant clinical randomized controlled trials (RCTs).

## 3. Methods

The current research has been registered on the Open Science Framework with DOI 10.17605/OSF.IO/78RCW. This protocol is reported based on the Preferred Reporting Items for Systematic Reviews and Meta-Analysis Protocol (PRISMA-P) statement guidelines.

### 3.1. Inclusion criteria for study selection

#### 3.1.1. Types of studies.

We intend to incorporate all RCTs that investigate adjuvant chemotherapy alone for advanced endometrial cancer. There will be no language or release status restrictions imposed.

#### 3.1.2. Types of participants.

All women with treated endometrial cancer will be included.

#### 3.1.3. Types of interventions.

In the experimental group, adjuvant chemotherapy should be used as the only mode of intervention. The use of adjuvant chemotherapy in conjunction with other treatments is not permitted. All types of treatments are permitted in the control group; however, no forms of adjuvant chemotherapy are permitted in the control group.

#### 3.1.4. Types of outcome measures.

The significant outcomes measured are the survival rate, the rate of symptom alleviation, the rate of tumor recurrence, and the rate of the emergence of a new malignancy. Adverse consequences are subsequent events that may occur.

### 3.2. Search Methods for identification of studies

#### 3.2.1. Electronic searches.

To establish all RCTs evaluating the efficacy and safety of adjuvant chemotherapy alone in patients with advanced endometrial carcinoma, a comprehensive systematic review and meta-analysis were conducted in PubMed, EMBASE, Cochrane Library, Web of Science, Wanfang, China National Knowledge Infrastructure (CNKI), and Chinese Biological Medical Database (CBM). Cancer, neoplasm, tumor, carcinoma, chemotherapy, and adjuvant chemotherapy were among the index words, which included the medical subject headings (MeSH) for endometrial neoplasms and uterine neoplasms as well as the text words endometrium and endometrial neoplasms, uterus and uterine neoplasms, and chemotherapy and adjuvant chemotherapy.

#### 3.2.2. Searching other resources.

We will examine the reference lists and citations of all RCTs and relevant systematic reviews discovered via electronic searches. Besides, we intend communicate with study authors to seek additionally published or unpublished data if necessary.

### 3.3. Data collection and analysis

#### 3.3.1. Selection of studies.

We will use 2 independent authors to identify relevant articles for inclusion during the title/abstract and full-text phases of the screening process. Titles that either of the authors has accepted will advance to the abstract screening stage during the title screening stage. The abstracts that either of the authors has authorized will continue to the full-text screening stage once accepted by the abstract screening stage. Abstracts that both authors have rejected will be disqualified. After the full-text screening, the records that both authors have authorized will be included in our evaluation. The writers will debate their differences until they achieve a consensus on resolving their differences.

#### 3.3.2. Data extraction and management.

Separately and independently, data from the chosen studies will be extracted by 2 writers, and a discussion with a third author will address any discrepancies between the authors. The following information will be collected from the study: the study’s identity, study characteristics, participant characteristics, and attributes of the intervention and comparator, among other things. EndNote X9 is used to handle the results of literature searches, and it will be utilized to delete duplicate data from the results. All of the information gathered is recorded in a Microsoft Excel spreadsheet.

#### 3.3.3. Assessment of risk of bias in included studies.

Utilizing the criteria provided in the *Cochrane Handbook for Systematic Reviews of Interventions*, 2 authors will evaluate the risk of bias in each trial in a separate and independent manner.^[14]^ The writers will debate their differences until they achieve a consensus on resolving their differences.

#### 3.3.4. Measures of treatment effect.

To analyze outcome measures for continuous data, mean differences (MDs) with 95% confidence intervals (CIs) will be utilized in conjunction with 95% CIs. The results would be combined on a log scale and then displayed on the original scale if continuous data were reported using geometric means. When dealing with binary data, the risk ratio with 95% CIs will be employed to assess outcome measures.

#### 3.3.5. Assessment of heterogeneity.

The *P* values and the *I²* statistic will be used to determine the degree of heterogeneity. Using the Mantel-Haenszel fixed-effects model, we will pool the data if *I²* ≤ 50%. In any other case, subgroup analysis or meta-regression will be employed to investigate the factors behind heterogeneity in the sample.

#### 3.3.6. Assessment of reporting bias.

Where we notice 10 or more articles, a funnel plots will be applied to evaluate if there is any reporting bias. The asymmetry of the funnel plot will be evaluated visually.

#### 3.3.7. Sensitivity analysis.

If required, we will eliminate trials with a high risk of bias to investigate the impact of these trials on the findings. In a sensitivity analysis, we will additionally look at the impact of assuming terrible outcomes for missing data.

## 4. Discussion

For patients with advanced endometrial cancer, the current research will consolidate all available outcome data to determine the efficacy of adjuvant chemotherapy alone in terms of survival. According to the outcomes of this research, adjuvant chemotherapy alone will give evidence as to whether or not it will deliver promising advantages in patients with advanced endometrial cancer. But the safety of adjuvant chemotherapy as a stand-alone treatment will also be evaluated. Furthermore, the strength of the results will be summarized via the Cochrane risk of bias tool, which will be assessed. The findings of this systematic review and meta-analysis will be helpful to information for doctors in making judgments in clinical practice, as well as for health policymakers in making decisions about health policy.

## Author contributions

Conceptualization: Jiayi Guo, Siqi Li.Data curation: Jiayi Guo, Siqi Li.Formal analysis: Jiayi Guo, Siqi Li.Investigation: Jiayi Guo, Siqi Li.Methodology: Jiayi Guo, Siqi Li, Yuping Wu.Software: Jiayi Guo.Supervision: Yuping Wu.Writing – original draft: Jiayi Guo.Writing – review & editing: Yuping Wu.
